# Suicidal Thoughts and Trajectories of Psychopathological and Behavioral Symptoms in Adolescence

**DOI:** 10.1001/jamanetworkopen.2023.53166

**Published:** 2024-01-25

**Authors:** Akito Uno, Daiki Nagaoka, Satoshi Usami, Satoshi Yamaguchi, Rin Minami, Riki Tanaka, Yutaka Sawai, Ayako Okuma, Syudo Yamasaki, Mitsuhiro Miyashita, Atsushi Nishida, Kiyoto Kasai, Shuntaro Ando

**Affiliations:** 1 Department of Neuropsychiatry, Graduate School of Medicine, The University of Tokyo, Tokyo, Japan; 2 Graduate School of Education, The University of Tokyo, Tokyo, Japan; 3 Research Center for Social Science and Medicine, Tokyo Metropolitan Institute of Medical Science, Tokyo, Japan

## Abstract

**Importance:**

The suicidal risk of psychopathology in adolescence is suggested to differ based on its longitudinal trajectory, but the comorbidity of these symptom trajectories has not been well examined. This study comprehensively clustered trajectories of multiple psychopathological and behavioral symptoms and examined their associations with suicidal thoughts in adolescence.

**Objective:**

To determine which categories and trajectories of psychopathological and behavioral symptoms are associated with suicidal thoughts in adolescence, accounting for comorbid symptoms.

**Design, Setting, and Participants:**

This population-based cohort study in Japan used data from the Tokyo Teen Cohort (TTC) study, which was established in 2012 and is currently ongoing. Data from 3 waves of surveys conducted at ages 10, 12, and 16 years from October 2012 to September 2021 were used. Of the adolescents in the cohort, participants with at least 2 evaluations of psychopathological and behavioral symptoms were included. Data were analyzed from December 2022 to March 2023.

**Exposure:**

Latent class growth analysis was used to cluster the trajectory of each psychopathological and behavioral symptom.

**Main Outcomes and Measures:**

The associations between symptom trajectories and suicidal thoughts at age 16 were examined. Suicidal thoughts were assessed using a self-report questionnaire. Psychopathological and behavioral symptoms were assessed using the 8 subscale scores of the caregiver-report Child Behavior Checklist.

**Results:**

This study included 2780 adolescents (1306 female participants [47.0%]). Of the 1920 adolescents with data on suicidal thoughts, 158 (8.2%) had suicidal thoughts. The median (IQR) age was 10.2 (10.0-10.3) years at the first evaluation, 11.9 (11.8-12.1) years at the second evaluation, and 16.3 (16.1-16.5) years at the last evaluation. The clustering pattern of trajectories varied depending on symptom categories. After adjusting for each symptom trajectory and confounders, adolescents with persistent high withdrawn symptoms (odds ratio [OR], 1.88; 95% CI, 1.10-3.21) and those with increasing somatic symptoms (OR, 1.97; 95% CI, 1.16-3.34) had a significantly higher risk of suicidal thoughts than adolescents without these symptoms. There was no interaction between these symptom trajectories and the risk of suicidal thoughts.

**Conclusions and Relevance:**

This cohort study found that persistent withdrawn symptoms and increasing somatic symptoms during early to midadolescence were associated with an increased risk of suicidal thoughts in midadolescence, even after accounting for comorbid symptoms and confounders. Attention should be paid to the suicidal risk associated with these symptoms, particularly when they persist or increase in the longitudinal follow-up.

## Key Points

**Question:**

Which categories and trajectories of psychopathological and behavioral symptoms are associated with suicidal thoughts in adolescence after accounting for comorbid symptoms?

**Findings:**

In this cohort study of 2780 adolescents, the clustering pattern of trajectories varied depending on the categories of psychopathological and behavioral symptoms. After adjusting for each symptom trajectory and confounders, adolescents with persistent withdrawn symptoms and those with increasing somatic symptoms had a significantly higher risk of suicidal thoughts than adolescents without these symptoms.

**Meaning:**

These findings suggest that to prevent adolescent suicide, it is important to pay attention to the risk of social withdrawal and somatic symptoms, particularly when these symptoms persist or increase in longitudinal follow-up.

## Teaser

This cohort study evaluates which categories and trajectories of psychopathological and behavioral symptoms are associated with suicidal thoughts among adolescence in Japan.

## Introduction

The importance of adolescent mental health has been increasingly recognized,^1,2^ and the World Health Organization has recommended mental health promotive and preventive interventions for all adolescents.^3^ However, about 10% to 20% of adolescents experience suicidal thoughts,^4,5^ and adolescent suicidal thoughts predict suicide attempts.^6^ Suicide remains one of the leading causes of death among adolescents worldwide,^7,8^ highlighting the need for further prevention. Psychopathology is a major risk factor for adolescent suicide,^7,9,10^ and many psychiatric disorders emerge during adolescence.^11,12^ Psychological autopsies, retrospective psychiatric evaluations of individuals who died by suicide, have shown that mood disorder (51%-76%) and substance use disorder (26%-62%) are 2 common diagnoses.^13,14^

Recent cohort studies have suggested that the risk of suicidal behaviors in adolescents with psychopathological and behavioral symptoms depends on the longitudinal trajectories of these symptoms. For example, depressive symptoms that persist from adolescence into adulthood, rather than being limited to adolescence, are associated with a higher risk of self-harm with suicidal thoughts in adulthood.^15^ It has also been proposed that boys with hyperactivity or inattention symptoms that persist moderately or highly from childhood to adolescence are at a greater risk of suicidal thoughts and attempts during adolescence than those with fewer symptoms trajectory.^16^ However, these previous studies have only examined the association of suicidal behaviors with a single category of symptoms.

Most adolescent psychopathological and behavioral symptoms are developmentally fluid^17,18^ and nonspecific to any psychiatric diagnosis.^19^ As a result, comorbidity is common and 20% to 50% of adolescents with psychiatric symptoms have more than 1 category of symptoms.^20,21^ Despite this, no studies that we know of have investigated the association of suicidal behaviors with trajectories of multiple psychopathological and behavioral symptoms. Therefore, it remains unknown which categories and trajectories of symptoms are associated with a higher risk of suicidal behaviors when dealing with comorbid symptoms. This study aims to comprehensively examine the trajectories of multiple psychopathological and behavioral symptoms and clarify which symptom trajectories are associated with suicidal thoughts.

## Methods

### Sample

We used data from the Tokyo Teen Cohort (TTC) study.^22^ TTC is a prospective birth cohort study of the general population that aims to examine mental and physical development during adolescence. Participants in the study were children born between September 2002 and August 2004 in 3 municipalities in Tokyo, Japan (Setagaya-ku, Mitaka-shi, and Chofu-shi). In the baseline survey, participants were randomly selected from the 18 830 eligible children using resident registers, as described elsewhere.^22^ See eMethods 1 in Supplement 1 for the flowchart of participant selection. A total of 3171 adolescents at age 10 years, 3007 at age 12 years (follow-up rate, 94.8%), and 2616 at age 16 years (follow-up rate, 82.5%) participated in the study. This study used data from 3 waves of data collection at ages 10, 12, and 16 years conducted from October 2012 to September 2021. Trained examiners visited participants’ homes at each time point and administered self-report questionnaires to the child and caregiver. At the first visit, written informed consent was obtained from the parents. Participants with available Child Behavior Checklist (CBCL) scores from at least 2 time points were included in this study.

TTC is a joint study by the Tokyo Metropolitan Institute of Medical Science, the University of Tokyo, and the Graduate University for Advanced Studies, and was approved by the ethics committees at each institution. This study followed the Strengthening the Reporting of Observational Studies in Epidemiology (STROBE) reporting guideline.

### Suicidal Thoughts

Participants were asked in the self-report questionnaire, “Do you currently think that you should not be alive?” when they were age 16. This question was adapted to capture passive suicidal thoughts that emerge in the early stages of the suicide spectrum.^23,24^ The answer choices were: no, somewhat no, somewhat yes, and yes. We dichotomized the participants into those without suicidal thoughts (answered no and somewhat no) and those with suicidal thoughts (answered yes and somewhat yes).

### Psychopathological and Behavioral Symptoms

Psychopathological and behavioral symptoms were measured using the Japanese version of the CBCL 4 to 18^25^ at 3 time points when participants were aged 10, 12, and 16 years. CBCL is an internationally used clinical and research instrument for comprehensively assessing children’s problems, which has also been validated in Japanese.^26^ The primary caregivers answered each item on a scale of 0 (not true), 1 (somewhat or sometimes true), or 2 (very true or often true). This study used the following 8 subscale scores: withdrawn (score range 0-18), somatic complaints (0-18), anxious depressed (0-28), social problems (0-16), thought problems (0-14), attention problems (0-22), delinquent behavior (0-26), and aggressive behavior (0-40). See eMethods 2 in Supplement 1 for the items included in each subscale.

### Confounders

Confounder selection was based on a disjunctive cause criterion, in which a variable that is the cause of either exposure or outcome is treated as a confounder.^27^ Based on previous studies on risk factors of psychopathology and suicidal thoughts,^7,9,10,28,29^ the following variables were treated as confounders: sex, annual household income, separation from primary caregiver, bereavement from family members, mental health problems of mother or father, alcohol consumption of mother or father, being bullied, and lifetime experience of suicidal thoughts. Lifetime experience of suicidal thoughts was assessed at age 12 years, and the other variables were assessed at age 10 years. See eMethods 3 in Supplement 1 for the details on the measures.

### Statistical Analysis

#### Latent Class Growth Analysis

To cluster psychopathological and behavioral symptom trajectories, we used latent class growth analysis (LCGA) on the 8 subscale scores of the CBCL, using Mplus version 8.8 (Muthén and Muthén).^30^ The scores were standardized and missing values were handled using the full information maximum likelihood method under the assumption of missing at random (eTable 1 in Supplement 1). Our model assumed linear change between time points, with constant residual variance over classes and time. More complex models did not converge. Models were created by optimization with the expectation maximization algorithm with 1000 random starting values. We increased the number of classes up to 6. Maximum likelihood with robust standard errors was used as the estimator. Model selection was primarily based on the Lo-Mendell-Rubin test (significance level set at 2-sided *P* < .05), which determines if the k-class model is superior to the k-1 class model. Akaike information criterion, bayesian information criterion (BIC), sample size adjusted BIC, entropy, size of the smallest class, and average posterior probability of assignment were also considered. To make interpretation easier, trajectory patterns were given names. We followed the Guidelines for Reporting on Latent Trajectory Studies (eAppendix 1 in Supplement 1).

#### Logistic Regression Analysis

To examine the association between symptom trajectories clustered by LCGA and suicidal thoughts, we performed logistic regression analysis on participants who had available data on suicidal thoughts. We fitted 4 models. Model 1 was a univariable logistic regression analysis examining the association of each symptom trajectory with suicidal thoughts. Model 2 was a multivariable logistic regression analysis including only symptoms that were statistically significant in Model 1. Model 3 added confounders to model 2. Model 4 was a multivariable logistic regression analysis including symptoms that were statistically significant in model 3, their interaction term, and confounders. Although we did not set any hypotheses in advance, we examined interaction to clarify if each symptom trajectory that was significant in model 3 was independently associated with suicidal thoughts. For the logistic regression analyses, we treated the trajectory subgroup with the largest proportion of each symptom as the reference. Missing values were handled using multiple imputation methods with the mice package.^31^ The imputation procedure included explanatory variables, outcome variables, and covariates. We created 100 data sets and combined them according to Rubin rules. The above analyses were performed using R version 4.2.1 (R Project for Statistical Computing). The significance level was set at *P* < .05.

To confirm the validity of the outcome dichotomization, we performed a sensitivity analysis where only participants who answered yes were considered to have suicidal thoughts. Additionally, we examined the cross-sectional association between symptoms and suicidal thoughts by using standardized CBCL subscale scores at age 16 years instead of symptom trajectories in the logistic regression analysis. Data were analyzed from December 2022 to March 2023.

## Results

A total of 2780 adolescents (1306 female participants [47.0%]) were included in this study. Of the 1920 adolescents with data on suicidal thoughts, 158 (8.2%) had suicidal thoughts. The median (IQR) age was 10.2 (10.0-10.3) years at the first evaluation, 11.9 (11.8-12.1) years at the second evaluation, and 16.3 (16.1-16.5) years at the last evaluation. The annual household income of the participants was ¥0 to ¥2.99 million for 4.4% (117 of 2677), ¥3 to ¥5.99 million for 24.6% (660 of 2677), ¥6 to ¥9.99 million for 40.5% (1085 of 2677), and more than ¥10 million for 30.4% (815 of 2677). A total of 35% (970 of 2771) of adolescents had experienced bereavement from family members. Mental health problems were reported for 4.8% (123 of 2561) of mothers and 3.5% (91 of 2573) of fathers. There were no significant differences in demographic characteristics and mean score of psychopathological and behavioral symptoms between the included and excluded adolescents (eTable 2 in Supplement 1). See eFigure 1 in Supplement 1 for the correlation between CBCL subscale scores. The CBCL subscale scores had missing values ranging from 6 to 38 (0.2%-1.4%) at age 10 years, 123-144 (4.4%-5.2%) at age 12 years, and 548-567 (19.7%-20.4%) at age 16 years. The rate of missing data on suicidal thoughts was 30.9% (860 of 2780). (Table 1)

**Table 1.** Descriptive Statistics of the Study Population

Characteristic	Participants, No./total No. (%)		
	Age, y		
	10	12	16
Sex			
Male	1474/2780 (53.0)	NA	NA
Female	1306/2780 (47.0)	NA	NA
Annual household income			
¥0 to ¥2.99 Million	117/2677 (4.4)	NA	NA
¥3 to ¥5.99 Million	6602677 (24.6)	NA	NA
¥6 to ¥9.99 Million	1085/2677 (40.5)	NA	NA
More than ¥10 Million	815/2677 (30.4)	NA	NA
Bereavement from family members	970/2771 (35.0)	NA	NA
Separation from primary caregiver	14/2779 (0.5)	NA	NA
Mental health problems of mother	123/2561 (4.8)	NA	NA
Mental health problems of father	91/2573 (3.5)	NA	NA
Alcohol consumption of mother			
Never	794/2572 (31.0)	NA	NA
Less than once a month	390/2572 (15.2)	NA	NA
2 to 4 times a month	460/2572 (17.9)	NA	NA
2 to 3 times a week	420/2572 (16.3)	NA	NA
More than 4 times a week	508/2572 (19.8)	NA	NA
Alcohol consumption of father			
Never	408/2522 (16.2)	NA	NA
Less than once a month	163/2522 (6.5)	NA	NA
2 to 4 times a month	295/2522 (11.7)	NA	NA
2 to 3 times a week	467/2522 (18.5)	NA	NA
More than 4 times a week	1189/2522 (47.1)	NA	NA
Bullied	876/2751 (31.8)	NA	NA
Lifetime experience of suicidal thoughts	NA	496/2425 (20.1)	NA
CBCL, mean (SD)			
Withdrawn	1.48 (1.71)	1.39 (1.82)	1.53 (2.02)
Somatic complaints	0.53 (1.10)	0.66 (1.21)	1.23 (1.93)
Anxious depressed	2.81 (3.12)	2.18 (2.85)	2.14 (3.12)
Social problems	2.32 (2.17)	1.29 (2.10)	1.44 (1.80)
Thought problems	0.52 (1.05)	0.44 (1.00)	0.50 (1.06)
Attention problems	3.41 (2.98)	2.96 (2.86)	2.76 (2.80)
Delinquent behavior	0.93 (1.30)	0.77 (1.34)	0.75 (1.40)
Aggressive behavior	4.06 (4.35)	3.35 (4.02)	2.54 (3.45)
Suicidal thoughts	NA	NA	158/1920 (8.2)

Abbreviations: CBCL, Child Behavior Checklist; NA, not applicable.

The symptom trajectories of the 8 CBCL subscale scores were clustered using LCGA (Figure). See eTable 3 in Supplement 1 for fit statistics and the estimated means of the models and the observed individual trajectories (eFigure 2 in Supplement 1).

**Figure.**
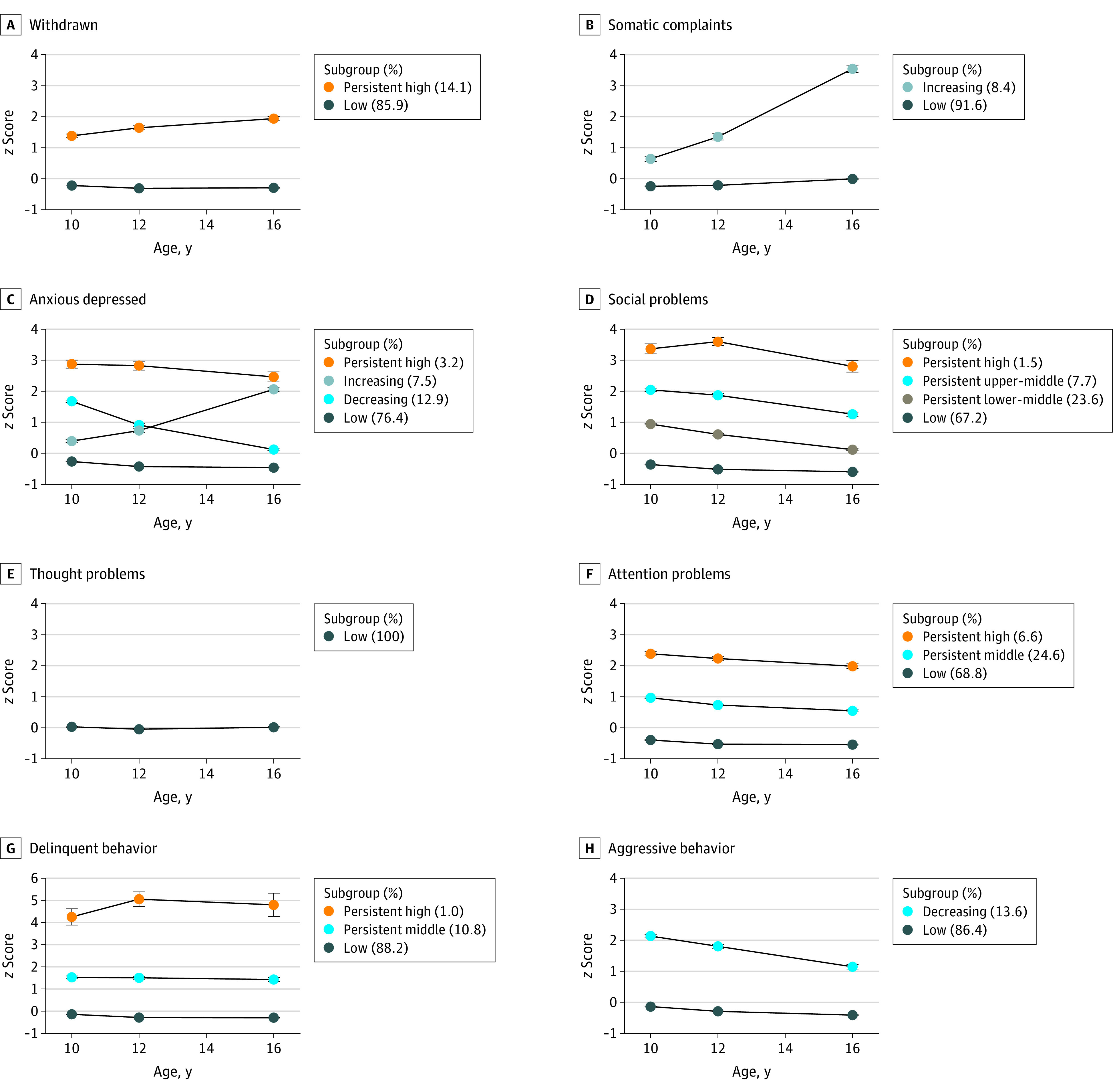
Trajectories of Psychopathological and Behavioral Symptoms The points represent the mean for standardized CBCL subscale scores per subgroup. Error bars indicate SE.

In the logistic regression analysis, we included data from 1920 participants whose data on suicidal thoughts were available. Among the symptom trajectories, thought problems with the 1-class solution were not included in the models. The low subgroup was used as a reference for all symptoms. In model 1, all symptoms except delinquent behavior were significantly associated with suicidal thoughts. In model 2, persistent high withdrawn (odds ratio [OR], 1.75; 95% CI, 1.05-2.90) and increasing somatic complaints (OR, 2.24; 95% CI, 1.37-3.36) were significantly associated with suicidal thoughts after adjusting for other symptom trajectories. In model 3, persistent high withdrawn (OR, 1.88; 95% CI, 1.10-3.21) and increasing somatic complaints (OR, 1.97, 95% CI, 1.16-3.34) remained significantly associated with suicidal thoughts after adjusting for other symptom trajectories and confounders. The prevalence of suicidal thoughts in adolescents with persistent high withdrawn was more than twice as high as those with low withdrawn (46 of 281 [16.4%] vs 112 of 1630 [6.9%], respectively). The prevalence of suicidal thoughts in adolescents with increasing somatic complaints was approximately 3 times as high as those with low somatic complaints (35 of 169 [20.7%] vs 123 of 1751 [7.0%], respectively) (eTable 4 in Supplement 1). In model 4, the interaction between persistent high withdrawn and increasing somatic complaints was not significant, while the association between these symptom trajectories and suicidal thoughts remained significant (Table 2). The variance inflation factors of the variables used in the models were all less than 2.0 (eTable 5 in Supplement 1), suggesting that multicollinearity was minimal. The results of the likelihood ratio test examining the overall effect of each symptom trajectory group are shown in eTable 6 in Supplement 1. In a sensitivity analysis of the outcome dichotomization, the results were similar to the main analyses (eAppendix 2 in Supplement 1). Additional analysis on the cross-sectional association between symptoms and suicidal thoughts at age 16 years revealed that, after adjusting for each symptom score and confounders, withdrawn (OR, 1.57; 95% CI, 1.28-1.92) and social problems (OR, 0.69; 95% CI, 0.51-0.93) had a significant cross-sectional association with suicidal thoughts, but not somatic complaints (OR, 1.10; 95% CI, 0.96-1.25) (eAppendix 3 in Supplement 1).

**Table 2.** Logistic Regression Analysis Examining the Association of Symptom Trajectories With Suicidal Thoughts at Age 16 Years

Symptom	Participants, %	Model 1		Model 2		Model 3		Model 4	
		OR (95% CI)	*P* value	OR (95% CI)	*P* value	OR (95% CI)	*P* value	OR (95% CI)	*P* value
Persistent high withdrawn	14.1	2.67 (1.84-3.86	<.001	1.75 (1.05-2.90)	.03	1.88 (1.10-3.21)	.02	1.89 (1.15-3.13)	.01
Increasing somatic complaints	8.4	3.46 (2.28-5.23)	<.001	2.24 (1.37-3.36)	.001	1.97 (1.16-3.34)	.01	2.26 (1.17-4.35)	.02
Decreasing anxious depressed	12.9	1.24 (0.75-2.05)	.40	0.96 (0.55-1.65)	.87	0.78 (0.43-1.39)	.40	NA	NA
Increasing anxious depressed	7.5	3.75 (2.44-5.77)	<.001	1.98 (1.15-3.43)	.01	1.5 (0.84-2.67)	.18	NA	NA
Persistent high anxious depressed	3.2	1.96 (0.87-4.44)	.11	0.78 (0.29-2.10)	.78	0.48 (0.16-1.39)	.17	NA	NA
Persistent lower-middle social problems	23.6	1.55 (1.08-2.22)	.02	1.07 (0.68-1.66)	.78	1.03 (0.64-1.65)	.90	NA	NA
Persistent upper-middle social problems	7.7	1.47 (0.80-2.72)	.21	0.67 (0.30-1.48)	.32	0.61 (0.26-1.44)	.26	NA	NA
Persistent high social problems	1.5	1.57 (0.46-5.28)	.47	0.56 (0.13-2.33)	.42	0.38 (0.082-1.79)	.22	NA	NA
Persistent middle attention problems	24.6	1.49 (1.03-2.15)	.04	1.05 (0.66-1.69)	.83	1.1 (0.66-1.81)	.72	NA	NA
Persistent high attention problems	6.6	2.27 (1.32-3.91)	.003	1.35 (0.61-3.00)	.46	1.39 (0.59-3.26)	.46	NA	NA
Persistent middle delinquent behavior	10.8	1.26 (0.77-2.06)	.36	NA	NA	NA	NA	NA	NA
Persistent high delinquent behavior	1	2.32 (0.66-8.10)	.19	NA	NA	NA	NA	NA	NA
Decreasing aggressive behavior	13.6	1.72 (1.13-2.61)	.01	1.14 (0.69-1.86)	.61	1.1 (0.65-1.86)	.73	NA	NA
Persistent high withdrawn × increasing somatic complaints	NA	NA	NA	NA	NA	NA	NA	0.82 (0.32-2.15)	.69

Abbreviations: NA, not applicable; OR, odds ratio.

Model 1 was a univariable logistic regression analysis. Model 2 was a multivariable logistic regression analysis including symptoms that were significant in Model 1. Model 3 added confounders to Model 2. Model 4 was a multivariable logistic regression analysis including symptoms that were significant in Model 3, their interaction term, and confounders. The low subgroup was used as a reference for all symptoms.

## Discussion

This is the first study we know of to comprehensively cluster the longitudinal trajectory of adolescent psychopathological and behavioral symptoms and examine their associations with suicidal thoughts in midadolescence. The clustering patterns of trajectories varied depending on the symptom categories. Among these symptom trajectories, persistent high withdrawn symptoms and increasing somatic symptoms were associated with an increased risk of suicidal thoughts in midadolescence. There was no interaction effect between these 2 trajectories on the risk of suicidal thoughts.

Persistent high withdrawn symptoms were shown to be associated with an elevated risk of suicidal thoughts in midadolescence, consistent with previous studies.^32,33^ Since the cross-sectional association between withdrawn symptoms and suicidal thoughts was also significant, caution is needed in interpreting the importance of the persistent trajectory itself. Although social withdrawal is complicated by many psychiatric disorders, including anxiety and phobic disorder and major depression,^34^ we found an independent association between withdrawn symptoms and suicidal thoughts. Social withdrawal is known to be associated with social isolation,^35^ and social isolation is associated with suicidal thoughts during adolescence.^36,37^ Therefore, the loss of protective social connections^9,38^ may explain the independent association between withdrawn symptoms and suicidal thoughts.

Increasing somatic symptoms were also found to be independently associated with suicidal thoughts in midadolescence, consistent with a systematic review on pain in adolescence which showed a significant association between pain and suicidality even after adjusting for depression.^39^ A previous cohort study also showed that the number of somatic symptoms in midadolescence was associated with the risk of suicide-related behaviors in adulthood.^40^ The increasing trajectory of somatic symptoms should be important because the cross-sectional association with suicidal thoughts was not significant. About 4 absolute CBCL subscale scores increased between age 10 and 16 years (eTable 7 in Supplement 1). Somatic symptoms in this study were probably functional, as indicated by the annotation without known medical cause on the CBCL questionnaires. Headache, fatigue, and stomachache were the most frequent symptoms and often coexisted among participants in this study (eFigure 3 in Supplement 1), which is consistent with the previous study.^41^ While there are many factors contributing to functional somatic symptoms,^42^ one hypothesis is somatization, which refers to the physical manifestation of psychological distress, particularly prevalent in cultures that inhibit emotional expression.^43^ In addition, pain and suicidality have been suggested to share common psychological mechanisms such as future orientation, psychological flexibility, and mental imagery in adults.^44^ These psychological processes may contribute to the independent association between somatic symptoms and suicidal thoughts found in this study.

We clustered the trajectory of anxious and depressive symptoms from early to midadolescence into 4 subgroups (high, increasing, decreasing, and low), which accords with previous studies.^45,46,47^ Only the trajectory of increasing anxious and depressive symptoms showed a higher risk of suicidal thoughts, which is also consistent with a previous study.^15^ However, the association between anxious depressive symptoms and suicidal thoughts became nonsignificant after adjusting for other symptoms and confounders. This result seems to be inconsistent with a previous cross-sectional study in which depression, among various risk factors including CBCL subscales, was found to predict suicidal thoughts and behaviors.^48^ The longitudinal nature of our study may partly explain this disparity, as we clustered anxious depressive symptoms into subgroups with different trajectories. Social problems unexpectedly were implied to have a protective effect against suicidal thoughts after adjusting for other symptoms and confounders in the cross-sectional analysis (eAppendix 3 in Supplement 1). One possible explanation is that approximately half of the items of the CBCL social problems were associated with childish social relationships (eg, prefers being with younger kids). These symptoms might reflect a certain social connection with others, which could be protective against social isolation, and consequently prevent suicidal thoughts. Attention problems and aggressive behaviors also showed an association with suicidal thoughts in the univariable logistic regression analysis, but this association became nonsignificant after adjusting for other symptoms and confounders. Given the comorbidity of these symptoms with other symptoms during adolescence,^34,41,49,50^ one interpretation of our result is that social withdrawal and somatic complaints are associated with relatively higher psychological distress than other symptoms. Another possibility is that adolescent psychological distress is more likely to be expressed somatically or behaviorally rather than emotionally, but further investigation is needed to confirm this.

As a clinical implication, attention should be paid to withdrawn and somatic symptoms to prevent adolescent suicide. The coexistence of these symptoms increases the risk of suicidal thoughts additively, as their interaction term was not significant. Based on the hypothetical mechanisms by which social withdrawal and somatic symptoms independently associate with suicidal thoughts, social connections and psychological support may be important in the care of adolescents with these symptoms. This is valuable for a wide range of people involved in adolescent health, as social withdrawal and common somatic symptoms are often more noticeable than emotional symptoms such as depression. Although social withdrawal and somatic symptoms may not receive sufficient clinical attention,^51,52^ our findings showed their crucial role in suicide prevention, particularly when they persist or increase in the longitudinal follow-up.

### Strength and Limitations

One strength of this study was its longitudinal and comprehensive data collection with a high follow-up rate. By following up with participants for over 6 years, we were able to reveal the multiple trajectories of adolescent psychopathological and behavioral symptoms. Additionally, we could confirm the external validity of clustering by comparing our results with previous studies on anxious and depressive symptoms.

However, there were several limitations to our study. First, due to the limited time points of data collection, the LCGA only converged with a strong assumption, thus the applied model might be too simple. For example, we were unable to include thought problems in the logistic regression analysis due to the 1-class solution. Future studies with more time points could reveal more complex trajectories. Second, we did not evaluate some important variables such as genetics,^53^ adverse childhood experiences,^54^ and information about social gender.^55^ Furthermore, the confounders were evaluated at age 10 years, thus the time-varying effects of the confounders remained unclear. Additionally, baseline suicidal thoughts were assessed at age 12 years instead of age 10 years due to data availability. Third, we did not use a validated tool that captures the full range of suicidal thoughts and behaviors; instead we assessed passive suicidal thoughts using a single questionnaire to reduce the psychological burden of participants. Additionally, the psychopathological and behavioral symptoms were reported by caregivers rather than self-reported. However, this approach is somewhat justified because most adolescents with suicidal thoughts are reluctant to seek help.^56^

## Conclusions

In this cohort study, we found that persistent high withdrawn symptoms and increasing somatic symptoms during early to midadolescence were associated with an elevated risk of suicidal thoughts in midadolescence, even after accounting for comorbid psychopathological and behavioral symptoms and confounders. A wide range of people involved in adolescent health should pay attention to the suicidal risk associated with these symptoms and consider the possibility of providing psychosocial support, particularly when the symptoms persist or increase in the longitudinal follow-up.

## Notes

Supplement 1. **eMethods 1. **Flowchart of Participant Selection **eAppendix 1. **Guidelines for Reporting on Latent Trajectory Studies (GRoLTS) Checklist: Guidelines for Reporting on Latent Trajectory Studies **eMethods 2. **Items of Each Child Behavior Checklist (CBCL) Subscale **eMethods 3. **Detailed Information on Measures **eTable 1.** Comparison Between Participants With and Without Missing Values in CBCL Scores **eTable 2. **Comparison Between Included and Excluded Participants **eFigure 1.** Correlations Between CBCL Subscale Scores **eTable 3.** Fit Statistics of Latent Class Growth Analysis (LCGA) **eTable 4. **The Proportion of Adolescents With Suicidal Thoughts for Each Symptom Trajectory **eFigure 2.** Estimated Means of the Models and the Observed Individual Trajectories **eTable 5.** The Variance Inflation Factor (VIF) of the Variables Used in Each Regression Model **eTable 6.** The Overall Effect of Each Symptom Trajectory Group **eAppendix 2.** Sensitivity Analysis of the Outcome Dichotomization **eAppendix 3. **Additional Analysis of the Cross-Sectional Relationship Between Symptoms and Suicidal Thoughts **eTable 7.** The Raw CBCL Subscale Scores for Each Symptom Trajectory **eFigure 3. **Trajectories of Each Somatic Complaint per Subgroup
eReferences

Supplement 2.
Data Sharing Statement
