# Supplementary material for: Suicidal Thoughts and Trajectories of Psychopathological and Behavioral Symptoms in Adolescence
Source: JAMA Netw Open. 2024 Jan 25;7(1):e2353166. doi: 10.1001/jamanetworkopen.2023.53166 (PMC10811562; doi:10.1001/jamanetworkopen.2023.53166)
Supplement: Supplement 1. — eMethods 1. Flowchart of Participant Selection eAppendix 1. Guidelines for Reporting on Latent Trajectory Studies (GRoLTS) Checklist: Guidelines for Reporting on Latent Trajectory Studies eMethods 2. Items of Each Child Behavior Checklist (CBCL) Subscale eMethods 3. Detailed Information on Measures eTable 1. Comparison Between Participants With and Without Missing Values in CBCL Scores eTable 2. Comparison Between Included and Excluded Participants eFigure 1. Correlations Between CBCL Subscale Scores eTable 3. Fit Statistics of Latent Class Growth Analysis (LCGA) eTable 4. The Proportion of Adolescents With Suicidal Thoughts for Each Symptom Trajectory eFigure 2. Estimated Means of the Models and the Observed Individual Trajectories eTable 5. The Variance Inflation Factor (VIF) of the Variables Used in Each Regression Model eTable 6. The Overall Effect of Each Symptom Trajectory Group eAppendix 2. Sensitivity Analysis of the Outcome Dichotomization eAppendix 3. Additional Analysis of the Cross-Sectional Relationship Between Symptoms and Suicidal Thoughts eTable 7. The Raw CBCL Subscale Scores for Each Symptom Trajectory eFigure 3. Trajectories of Each Somatic Complaint per Subgroup eReferences [file jamanetwopen-e2353166-s001.pdf]

Supplemental Online Content

Uno A, Nagaoka D, Usami S, et al. Suicidal thoughts and trajectories of psychopathological and behavioral symptoms in adolescence. *JAMA Netw Open*. 2024;7(1):e2353166. doi:10.1001/jamanetworkopen.2023.53166

eMethods 1. Flowchart of Participant Selection
eAppendix 1. Guidelines for Reporting on Latent Trajectory Studies (GRoLTS) Checklist: Guidelines for Reporting on Latent Trajectory Studies
eMethods 2. Items of Each Child Behavior Checklist (CBCL) Subscale
eMethods 3. Detailed Information on Measures
eTable 1. Comparison Between Participants With and Without Missing Values in CBCL Scores
eTable 2. Comparison Between Included and Excluded Participants
eFigure 1. Correlations Between CBCL Subscale Scores
eTable 3. Fit Statistics of Latent Class Growth Analysis (LCGA)
eTable 4. The Proportion of Adolescents With Suicidal Thoughts for Each Symptom Trajectory
eFigure 2. Estimated Means of the Models and the Observed Individual Trajectories
eTable 5. The Variance Inflation Factor (VIF) of the Variables Used in Each Regression Model
eTable 6. The Overall Effect of Each Symptom Trajectory Group
eAppendix 2. Sensitivity Analysis of the Outcome Dichotomization
eAppendix 3. Additional Analysis of the Cross-Sectional Relationship Between Symptoms and Suicidal Thoughts
eTable 7. The Raw CBCL Subscale Scores for Each Symptom Trajectory
eFigure 3. Trajectories of Each Somatic Complaint per Subgroup
eReferences

This supplemental material has been provided by the authors to give readers additional information about their work.

© 2024 Uno A et al. *JAMA Network Open.*

eAppendix 1. STROBE Statement—Checklist of items that should be included in reports of *cohort studies*

	Item No	Recommendation	Page No
**Title and abstract**	1	(*a*) Indicate the study's design with a commonly used term in the title or the abstract	1
		(*b*) Provide in the abstract an informative and balanced summary of what was done and what was found	2
**Introduction**			
Background/rationale	2	Explain the scientific background and rationale for the investigation being reported	5-6
Objectives	3	State specific objectives, including any prespecified hypotheses	6
**Methods**			
Study design	4	Present key elements of study design early in the paper	6
Setting	5	Describe the setting, locations, and relevant dates, including periods of recruitment, exposure, follow-up, and data collection	6-7
Participants	6	(*a*) Give the eligibility criteria, and the sources and methods of selection of participants. Describe methods of follow-up	6-7, S4
		(*b*) For matched studies, give matching criteria and number of exposed and unexposed	-
Variables	7	Clearly define all outcomes, exposures, predictors, potential confounders, and effect modifiers. Give diagnostic criteria, if applicable	7-8
Data sources/ measurement	8*	For each variable of interest, give sources of data and details of methods of assessment (measurement). Describe comparability of assessment methods if there is more than one group	S5-8
Bias	9	Describe any efforts to address potential sources of bias	15-16
Study size	10	Explain how the study size was arrived at	S4
Quantitative variables	11	Explain how quantitative variables were handled in the analyses. If applicable, describe which groupings were chosen and why	8-10
Statistical methods	12	(*a*) Describe all statistical methods, including those used to control for confounding	8-10
		(*b*) Describe any methods used to examine subgroups and interactions	8-10
		(*c*) Explain how missing data were addressed	8-10
		(*d*) If applicable, explain how loss to follow-up was addressed	8-10
		(*e*) Describe any sensitivity analyses	8-10

© 2024 Uno A et al. *JAMA Network Open.*

eAppendix 1. STROBE Statement—Checklist of items that should be included in reports of *cohort studies* (continued)

	Item No	Recommendation	Page No
**Results**			
Participants	13*	(a) Report numbers of individuals at each stage of study—eg numbers potentially eligible, examined for eligibility, confirmed eligible, included in the study, completing follow-up, and analysed	10, 11
		(b) Give reasons for non-participation at each stage	10, 11
		(c) Consider use of a flow diagram	S4
Descriptive data	14*	(a) Give characteristics of study participants (eg demographic, clinical, social) and information on exposures and potential confounders	10, 25
		(b) Indicate number of participants with missing data for each variable of interest	10, 25
		(c) Summarise follow-up time (eg, average and total amount)	-
Outcome data	15*	Report numbers of outcome events or summary measures over time	10, 25
Main results	16	(*a*) Give unadjusted estimates and, if applicable, confounder-adjusted estimates and their precision (eg, 95% confidence interval). Make clear which confounders were adjusted for and why they were included	10-12, 26-27
		(*b*) Report category boundaries when continuous variables were categorized	-
		(*c*) If relevant, consider translating estimates of relative risk into absolute risk for a meaningful time period	-
Other analyses	17	Report other analyses done—eg analyses of subgroups and interactions, and sensitivity analyses	11-12, S20-21
**Discussion**			
Key results	18	Summarise key results with reference to study objectives	11
Limitations	19	Discuss limitations of the study, taking into account sources of potential bias or imprecision. Discuss both direction and magnitude of any potential bias	15-16
Interpretation	20	Give a cautious overall interpretation of results considering objectives, limitations, multiplicity of analyses, results from similar studies, and other relevant evidence	12-15
Generalisability	21	Discuss the generalisability (external validity) of the study results	12-15
**Other information**			
Funding	22	Give the source of funding and the role of the funders for the present study and, if applicable, for the original study on which the present article is based	18

*Give information separately for exposed and unexposed groups.

eMethods 1. Flowchart of participant selection

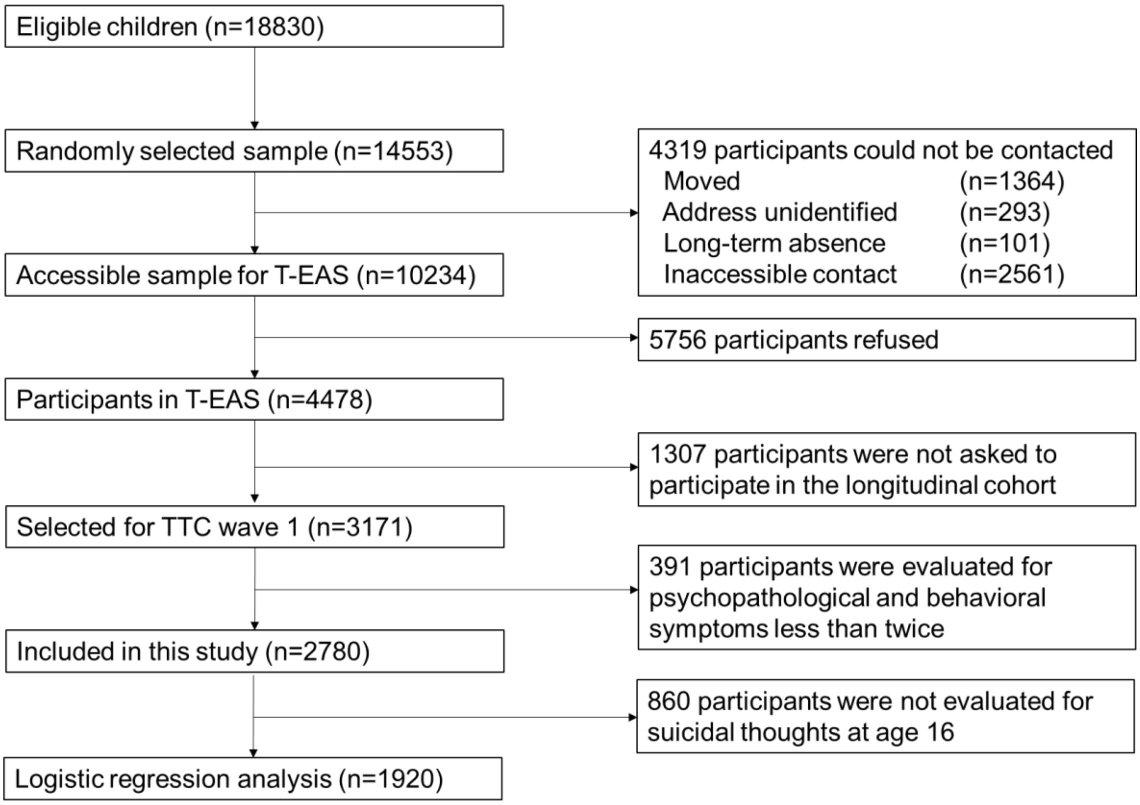

T-EAS: Tokyo Early Adolescence Survey (the baseline survey of TTC), TTC: Tokyo Teen Cohort study.

© 2024 Uno A et al. *JAMA Network Open.*

eMethods 2. Items of each Child Behavior Checklist (CBCL) subscale

Subscale	Item
Withdrawn	Would rather be alone than with others
	Refuses to talk
	Secretive, keeps things to self
	Too shy or timid
	Stares blankly
	Sulks a lot
	Underactive, slow moving, or lacks energy
	Unhappy, sad, or depressed
	Withdrawn, dosen't get involved with others
Somatic complaints	Feels dizzy or lightheaded
	Overtired without good reason
	Physical problems without know medical cause:
	Aches or pains (not stomach or headaches)
	Headaches
	Nausea, feels sick
	Problems with eyes (not if corrected by glasses)
	Rashes or other skin problems
	Stomachaches
	Vomiting, throwing up
Anxious depressed	Complains of loneliness
	Cries a lot
	Fears he/she might think or do something bad
	Feels he/she has to be perfect
	Feels or complains that no one loves him/her
	Feels others are out to get him/her
	Feels worthless or inferior
	Nervous, high strung, or tense
	Too fearful or anxious
	Feels too guilty
	Self-conscious or easily embarrassed
	Suspicious
	Unhappy, sad, or depressed
	Worries
Social problems	Acts too young for his/her age
	Clings to adults or too dependent
	Doesn't get along with other kids
	Gets teased a lot
	Not liked by other kids
	Overweight
	Poorly coordinated or clumsy
	Prefers being with younger kids
Thought problems	Can't get his/her mind off certain thoughts; obsessions
	Hears sound or voices that aren't there
	Repeats certain acts over and over; compulsions
	Sees things that aren't there
	Stares blankly

© 2024 Uno A et al. *JAMA Network Open.*

eMethods 2. Items included in each CBCL subscale (continued)

Subscale	Item
Thought problems	Strange behavior
	Strange ideas
Attention problems	Acts too young for his/her age
	Can't concentrate, can't pay attention for long
	Can't sit still, restless, or hyperactive
	Confused or seems to be in a fog
	Daydreams or gets lost in his/her thoughts
	Impulsive or acts without thinking
	Nervous, highstrung, or tense
	Nervous movements or twitching
	Poor school work
	Poorly coordinated or clumsy
	Stares blankly
Delinquent behavior	Doesn't seem to feel guilty after misbehaving
	Hangs around with others who get in trouble
	Lying or cheating
	Prefers being with other kids
	Runs away from home
	Sets fires
	Steals at home
Delinquent behavior	Steals outside the home
	Swearing or obscene language
	Thinks about sex too much
	Truancy, skips school
	Uses drugs for nonmedical purposes (don't include alcohol or tobacco)
	Vandalism
Aggressive behavior	Argues a lot
	Bragging, boasting
	Cruelty, bullying, or meanness to others
	Demands a lot of attention
	Destroys his/her own things
	Destroys things belonging to his/her family or others
	Disobedient at home
	Disobedient at school
	Easily jealous
	Gets in many fights
	Physically attacks people
	Screams a lot
	Showing off or clowning
	Stubborn, sullen, or irritable
	Sudden changes in mood or feelings
	Talks to much
	Teases a lot
	Temper tantrums or hot temper
	Threatens people
	Unusually loud

© 2024 Uno A et al. *JAMA Network Open.*

eMethods 3. Detailed information on Measures

sex

The primary caregiver answered male or female when the participant was at age 10.

annual household income

The primary caregivers answered 0 to 0.99, 1 to 1.99, 2 to 2.99, 3 to 3.99, 4 to 4.99, 5 to 5.99, 6 to 6.99, 7 to 7.99, 8 to 8.99, 9 to 9.99 or more than 10 million yen when the participant was at age 10. The responses were then grouped into 0 to 2.99, 3 to 5.99, 6 to 9.99, and more than 10 million yen when the participant was age 10.

separation from primary caregiver

The primary caregivers were asked the question 'Has your child ever lived away from you (primary caregiver) for more than one year?' They then answered yes or no when the adolescents were age 10.

bereavement from family members

The primary caregivers were asked the question 'Has a family member (mother, father, siblings, and grandparents) passed away since your child was born?' They then answered yes or no when the adolescents were age 10.

mental health problems of mother/father

The primary caregivers were asked the question ‘Have you experienced any physical or mental health problems that have lasted for more than one year? (This includes those that are expected to last more than one year in the future.)’ If they answered yes, they indicated the specific type of problem when the adolescents were age 10. Those who indicated ‘mental health problems’ were considered positive. The primary caregivers answered the same questions for both themselves and their partner, and the results were labeled as either ‘mental health problems of mother’ or ‘mental health problems of father’, depending on whether the primary caregiver was the mother or the father. Here, mental health problems of mother/father reflect both the family system and the genetic perspective, which cannot be separated.

alcohol consumption of mother/father

The primary caregivers were asked the question 'How many days a week do you drink alcohol?' They then answered ‘never’, ‘less than once a month’, ‘two to four times a month’, ‘two to three times a week’, ‘more than four times a week’, or ‘don't know/don't want to answer’ when the adolescents were age 10. The answer 'don't know/don't want to answer' was considered as a missing value.

The primary caregivers answered the same questions for both themselves and their partner, and the results

© 2024 Uno A et al. *JAMA Network Open.*

were labeled as either 'mental health problems of mother' or 'mental health problems of father', depending on whether the primary caregiver was the mother or the father."

bullied

Because children and their caregivers often report differently about children's experiences of being bullied[1,2], we considered it a negative only if both the primary caregivers and the adolescents answered "none. Otherwise, it was considered a positive. The primary caregivers were asked the question 'In the past two months, has your child been bullied by other children?' They then answered ‘several times a week’, ‘about once a week’, ‘two to three times a month’, ‘once or twice every two months’, or ‘none’ when the adolescents were age 10. The adolescents were also asked the questions 'In the past two months, have you been bullied by other children in your school?' and 'In the past two months, have you been bullied by other children in your school?' They then selected ‘several times a week’, ‘about once a week’, ‘two to three times a month’, ‘once or twice every two months’, or ‘none’ when they were age 10.

lifetime experience of suicidal thoughts at age 12

The adolescents were asked the question ‘I have ever thought I should not be alive.’ They then answered ‘strongly agree,’ ‘almost agree’, ‘almost disagree’, and ‘strongly disagree’ when they were at age 12. We dichotomized the participants into those without a lifetime experience of suicidal thoughts (answered ‘strongly disagree’ and ‘almost disagree’) and those with a lifetime experience of suicidal thoughts (answered ‘strongly agree’ and ‘almost agree’).

© 2024 Uno A et al. *JAMA Network Open.*

eTable 1. Comparison between participants with and without missing values in CBCL scores

	Participants with missing values in CBCL scores (n = 1838)	Participants without missing values in CBCL scores (n = 942)	*p*-value
sex			
male	990/1838 (53.9%)	484/942 (51.4%)	0.23
female	848/1838 (46.1%)	458/942 (48.6%)	
annual household income			
0 to 2.99 million yen	80/1775 (4.5%)	37/902 (4.1%)	0.053
3 to 5.99 million yen	409/1775 (23.0%)	251/902 (27.8%)	
6 to 9.99 million yen	740/1775 (41.7%)	345/902 (38.2%)	
more than 10 million yen	546/1775 (30.8%)	269/902 (29.8%)	
bereavement from primary caregiver	650/1834 (35.4%)	320/937 (34.2%)	0.53
separation from family members	11/1837 (0.6%)	3/942 (0.3%)	0.48
mental health problem of mother	73/1704 (4.3%)	50/857 (5.8%)	0.10
mental health problem of father	64/1712 (3.7%)	27/861 (3.1%)	0.50
alcohol consumption of mother			
never	531/1714 (31.0%)	263/858 (30.7%)	0.93
less than once a month	259/1714 (15.1%)	131/858 (15.3%)	
two to four times a month	299/1714 (17.4%)	161/858 (18.8%)	
two to three times a week	285/1714 (16.6%)	135/858 (15.7%)	
more than four times a week	340/1714 (19.8%)	168/858 (19.6%)	
alcohol consumption of father			
never	265/1680 (15.8%)	143/842 (17.0%)	0.82
less than once a month	107/1680 (6.4%)	56/842 (6.7%)	
two to four times a month	197/1680 (11.7%)	98/842 (11.6%)	
two to three times a week	321/1680 (19.1%)	146/842 (17.3%)	
more than four times a week	790/1680 (47.0%)	399/842 (47.4%)	
bullied	575/1823 (31.5%)	301/928 (32.4%)	0.67
lifetime experience of suicidal thoughts at age 12	339/1690 (20.1%)	157/735 (21.4%)	0.50
suicidal thoughts	125/1583 (7.9%)	33/337 (9.8%)	0.30

Values are No./total No. (%). We used the chi-square test for categorical variables to compare participant characteristics between participants between those with and without missing values in CBCL scores. No statistically significant differences were found between the two groups.

© 2024 Uno A et al. *JAMA Network Open.*

eTable 2. Comparison between included and excluded participants

Characteristic	Excluded (n=391)	Included (n=2780)	*p*-value
sex			
male	210/391 (53.7%)	1474/2780 (53.0%)	0.84
female	181/391 (46.3%)	1306/2780 (47.0%)	
Withdrawn	1.48 (1.71)	1.48 (1.71)	0.95
Somatic complaints	0.55 (1.25)	0.53 (1.10)	0.78
Anxious depressed	2.91 (2.98)	2.81 (3.12)	0.53
Social problems	2.56 (2.28)	2.32 (2.17)	0.050
Thought problems	0.58 (1.17)	0.52 (1.05)	0.31
Attention problems	3.46 (2.89)	3.41 (2.98)	0.77
Delinquent behavior	0.97 (1.33)	0.93 (1.30)	0.52
Aggressive behavior	4.39 (4.37)	4.06 (4.35)	0.17
annual household income			
0 to 2.99 million yen	25/369 (6.8%)	117/2677 (4.4%)	0.08
3 to 5.99 million yen	103/369 (27.9%)	660/2677 (24.7%)	
6 to 9.99 million yen	139/369 (37.7%)	1085/2677 (40.5%)	
more than 10 million yen	102/369 (27.6%)	815/2677 (30.4%)	
bereavement from primary caregiver	139/391 (35.5%)	979/2771 (35.3%)	0.88
separation from family members	2/391 (0.5%)	14/2779 (0.5%)	>0.99
mental health problem of mother	13/341 (3.8%)	123/2561 (4.8%)	0.50
mental health problem of father	11/341 (3.2%)	91/2573 (3.5%)	0.89
alcohol consumption of mother			
never	113/343 (3.9%)	794/2572 (30.9%)	0.24
less than once a month	37/343 (10.8%)	390/2572 (15.2%)	
2 to 4 times a month	70/343 (20.4%)	460/2572 (17.9%)	
2 to 3 times a week	56/343 (16.3%)	420/2572 (16.3%)	
more than 4 times a week	67/343 (19.5%)	508/2572 (19.8%)	
alcohol consumption of father			
never	44/329 (13.4%)	408/2522 (16.2%)	0.52
less than once a month	20/329 (6.1%)	163/2522 (6.5%)	
2 to 4 times a month	42/329 (12.8%)	295/2522 (11.7%)	
2 to 3 times a week	55/329 (16.7%)	467/2522 (18.5%)	
more than 4 times a week	168/329 (51.1%)	1189/2522 (47.1%)	
bullied	133/383 (34.7%)	876/2761 (31.7%)	0.28
lifetime experience of suicidal thoughts at age 12	22/81 (27.2%)	496/2425 (20.5%)	0.18
suicidal thoughts	2/20 (10.0%)	158/1920 (8.2%)	>0.99

Values are No./total No. (%) or mean (SD). We used the chi-square test for categorical variables and the t-test for continuous variables to compare participant characteristics between those included and excluded in the study. No statistically significant differences were found between the two groups.

© 2024 Uno A et al. *JAMA Network Open.*

eFigure 1. Correlations between CBCL subscale scores

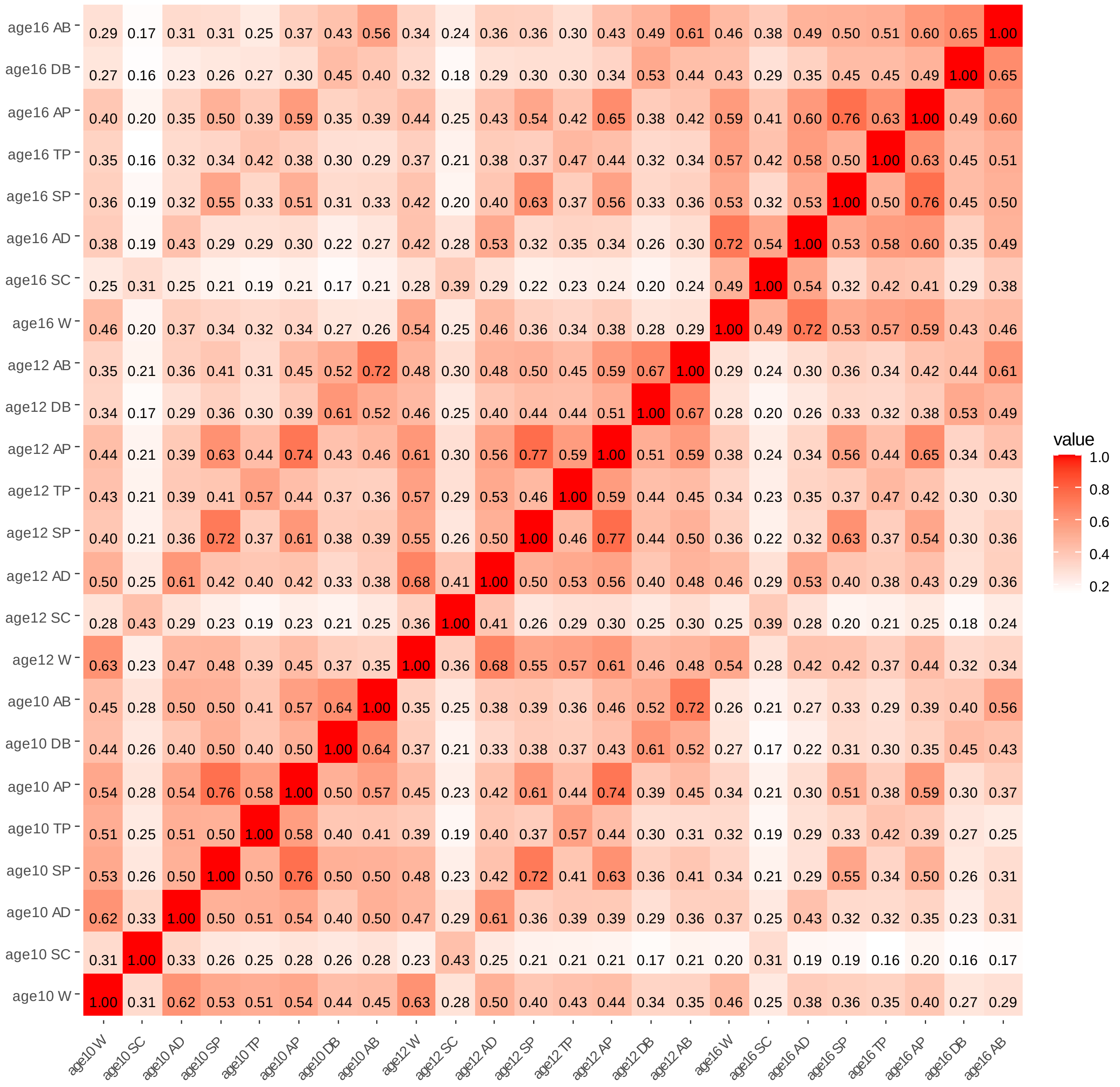

Values are Pearson's correlation coefficients. CBCL subscale scores were standardized.

W: withdrawn, SC: somatic complaints, AD: anxious depressed, SP: social problems, TP: thought problems, AP: attention problems, DB: delinquent behavior, AB: aggressive behavior

© 2024 Uno A et al. *JAMA Network Open.*

eTable 3. Fit statistics of latent class growth analysis (LCGA)

Symptoms	Number of classes	LL	AIC	BIC	aBIC	Entropy	LMR test *p* value	proportion per class (%)	APPA
Withdrawn	1	-10865	21736	21753	21744	NA	NA	100	100
	**2**	**-9608**	**19228**	**19264**	**19245**	**0.91**	**<0.0001**	**85.9: 14.1**	**98.2: 92.8**
	3	-9306	18629	18683	18654	0.92	0.30	83.2: 13.0: 3.8	97.9: 88.4: 91.7
	4	-9023	18070	18141	18103	0.89	0.0056	77.7: 12.8: 6.6: 2.9	96.5: 82.4: 88.4: 92.5
	5	-8905	17840	17929	17881	0.88	0.15	75.4: 12.1: 6.4: 3.6: 2.5	96.0: 80.4: 79.9: 85.7: 92.1
	6	-8796	17627	17734	17677	0.87	0.10	72.6: 13.6: 5.9: 3.7: 3.4: 0.8	94.5: 78.5: 82.5: 87.5: 87.3: 94.7
Somatic complaints	1	-10614	21235	21252	21243	NA	NA	100	100
	**2**	**-9403**	**18817**	**18853**	**18834**	**0.94**	**0.0009**	**91.6: 8.4**	**99.1: 90.2**
	3	-9051	18121	18174	18146	0.95	0.071	89.1: 7.7: 3.2	98.7: 91.0: 89.8
	4	-8645	17314	17385	17347	0.93	0.044	83.9: 10.4: 3.7: 2.0	97.5: 87.4: 91.4: 94.5
	5	-8461	16952	17041	16993	0.92	0.18	80.9: 10.2: 4.3: 4.0: 0.6	97.0: 85.8: 88.8: 88.9: 96.8
	6	-8322	16680	16786	16729	0.92	0.71	79.2: 10.3: 5.3: 3.7: 1.0: 0.5	97.1: 86.8: 84.1: 90.0: 92.8: 93.4
Anxious depressed	1	-10815	21637	21655	21645	NA	NA	100	100
	2	-9599	19210	19245	19226	0.90	<0.0001	85.0: 15.0	98.1: 92.2
	3	-9265	18548	18601	18572	0.89	0.048	78.1: 18.2: 3.7	96.7: 87.9: 92.7
	**4**	**-8913**	**17850**	**17921**	**17883**	**0.90**	**0.0007**	**76.4: 12.9: 7.5: 3.2**	**97.2: 86.2: 85.3: 92.9**
	5	-8772	17575	17664	17616	0.91	0.24	75.0: 12.8: 8.1: 3.1: 1.1	96.7: 85.7: 86.0: 90.3: 91.4
	6	-8684	17403	17510	17453	0.91	0.21	73.8: 13.0: 8.0: 3.1: 1.2: 0.9	96.6: 85.3: 84.1: 85.6: 90.7: 92.7
Social problems	1	-10725	21456	21474	21464	NA	NA	100	100
	2	-9265	18542	18577	18558	0.90	<0.0001	83.1: 16.9	98.2: 92.1
	3	-8788	17594	17647	17618	0.88	<0.0001	72.3: 22.9: 4.8	96.3: 88.9: 95.2
	**4**	**-8641**	**17306**	**17377**	**17339**	**0.86**	**0.020**	**67.2: 23.6: 7.7: 1.5**	**94.9: 84.5: 89.0: 94.7**
	5	-8532	17093	17182	17135	0.86	0.75	64.5: 24.4: 4.9: 4.5: 1.8	95.2: 81.7: 81.1: 84.8: 93.2
	6	s	16942	17049	16992	0.86	0.050	66.3: 20.3: 5.4: 5.6: 2.9: 1.5	94.4: 81.9: 82.0: 77.6: 82.6: 92.9

© 2024 Uno A et al. *JAMA Network Open.*

eTable 3. Fit statistics of latent class growth analysis (LCGA) (continued)

Symptoms	Number of classes	LL	AIC	BIC	aBIC	Entropy	LMR test p value	proportion per class (%)	APPA
Thought problems	**1**	**-10853**	**21712**	**21730**	**21720**	**NA**	**NA**	**100**	**100**
	2	-9374	18759	18795	18776	0.98	0.088	94.7: 5.3	99.6: 93.9
	3	-8905	17828	17882	17853	0.97	0.12	91.5: 6.9: 1.5	98.6: 95.3: 96.5
	4	-8549	17122	17193	17155	0.96	0.054	89.6: 5.1: 4.0: 1.3	98.9: 86.3: 93.9: 98.3
	5	-8377	16784	16873	16825	0.95	0.73	86.7: 7.2: 3.4: 1.7: 1.0	98.5: 83.7: 88.2: 93.0: 97.5
	6	-8240	16515	16622	16565	0.95	0.089	85.2: 6.2: 5.1: 1.6: 1.0: 0.9	97.9: 83.1: 87.2: 95.8: 93.8: 97.1
Attention problems	1	-10801	21607	21625	21615	NA	NA	100	100
	2	-9303	18618	18653	18634	0.90	<0.0001	81.0: 19.0	98.0: 93.0
	**3**	**-8914**	**17847**	**17900**	**17872**	**0.85**	**0.0050**	**68.8: 24.6: 6.6**	**95.5: 87.7: 92.3**
	4	-8765	17555	17626	17588	0.82	0.26	61.0: 25.6: 11.3: 2.0	93.0: 82.9: 86.1: 92.6
	5	-8609	17249	17338	17290	0.83	0.0017	58.0: 26.8: 7.6: 5.1: 2.5	92.8: 82.5: 82.6: 82.7: 91.3
	6	-8546	17128	17234	17177	0.82	0.41	56.5: 25.6: 8.2: 5.1: 3.6: 1.0	91.1: 80.7: 80.6: 79.9: 85.2: 89.8
Delinquent behavior	1	-10823	21651	21669	21660	NA	NA	100	100
	2	-9561	19133	19169	19150	0.97	0.032	94.1: 5.9	99.4: 94.7
	**3**	**-9114**	**18245**	**18299**	**18270**	**0.95**	**0.021**	**88.2: 10.8: 1.0**	**98.5: 90.2: 99.9**
	4	-8831	17686	17757	17719	0.94	0.27	85.6: 8.7: 4.7: 1.0	98.1: 87.4: 83.6: 98.7
	5	-8647	17324	17413	17365	0.93	0.46	82.3: 10.6: 4.3: 2.1: 0.6	97.2: 85.9: 85.3: 92.7: 98.9
	6	-8504	17044	17151	17093	0.92	0.12	80.8: 9.8: 5.5: 2.2: 1.0: 0.6	96.7: 84.1: 82.4: 92.4: 97.6: 98.1
Aggressive behavior	1	-10715	21436	21454	21444	NA	NA	100	100
	**2**	**-9175**	**18363**	**18398**	**18379**	**0.93**	**<0.0001**	**86.4: 13.6**	**98.7: 93.7**
	3	-8693	17403	17457	17428	0.91	0.11	77.2: 19.3: 3.5	97.4: 89.8: 94.1
	4	-8474	16972	17044	17005	0.88	0.034	71.3: 20.3: 7.1: 1.2	95.8: 84.9: 89.6: 96.2
	5	-8276	16582	16671	16624	0.88	0.025	69.3: 19.7: 5.0: 4.8: 1.1	95.5: 83.3: 87.4: 84.5: 98.9
	6	-8187	16410	16517	16459	0.88	0.042	69.1: 18.0: 4.3: 3.8: 3.7: 1.1	95.8: 82.7; 84.5: 76.7: 86.8: 99.7

NA: not applicable, LL: log-likelihood, AIC: Akaike information criterion, BIC: Bayesian information, aBIC: simple size adjusted BIC, LMR: Lo-Mendell-Rubin, APPA: the average posterior probability of assignment

© 2024 Uno A et al. *JAMA Network Open.*

eFigure 1. Estimated means of the models and the observed individual trajectories

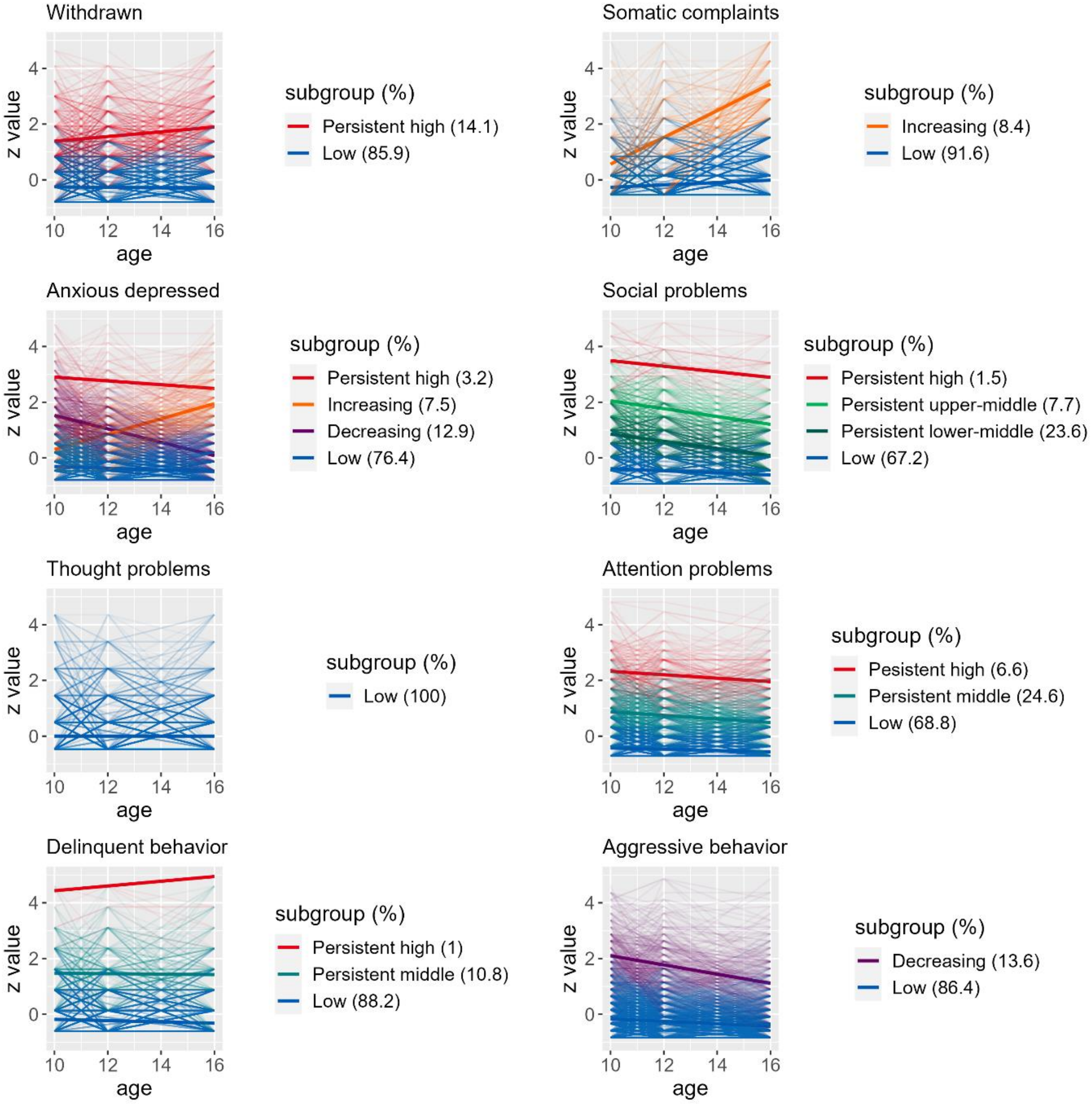

The bold lines represent the estimated trajectories of LCGA, while the background thin lines depict the trajectories of each individual, colored by subgroup membership.

© 2024 Uno A et al. *JAMA Network Open.*

eTable 4. The proportion of adolescents with suicidal thoughts for each symptom trajectory

Symptom	Trajectory	Suicidal thoughts
Withdrawn	Low (n=2387)	112/1639 (6.8%)
	Persistent high (n=393)	46/281 (16.4%)
Somatic complaints	Low (n=2547)	123/1751 (7.0%)
	Increasing (n=233)	35/169 (20.7%)
Anxious depressed	Low (n=2124)	97/1456 (6.7%)
	Decreasing (n=360)	20/246 (8.1%)
	Increasing (n=208)	34/161 (21.1%)
	Persistent high (n=88)	7/57 (12.3%)
Social problems	Low (n=1869)	92/1292 (7.1%)
	Persistent lower-middle (n=655)	50/472 (10.6%)
	Persistent upper-middle (n=213)	13/128 (10.2%)
	Persistent high (n=43)	3/28 (10.7%)
Thought problems	Low (n=2780)	158/1920 (8.2%)
Attention problems	Low (n=1912)	94/1341 (7.0%)
	Persistent middle (n=685)	46/456 (10.1%)
	Persistent high (n=183)	18/123 (14.6%)
Delinquent behavior	Low (n=2452)	135/1698 (8.0%)
	Persistent middle (n=301)	20/204 (9.8%)
	Persistent high (n=27)	3/18 (16.6%)
Aggressive behavior	Low (n=2402)	127/1670 (7.6%)
	Decreasing (n=378)	31/250 (12.4%)

Values were No./total No. (%)

© 2024 Uno A et al. *JAMA Network Open.*

eTable 5. The variance inflation factor (VIF) of the variables used in each regression model

Variable	Model 2	Model 3	Model 4
sex	NA	1.1	1.0
annual household income	NA	1.0	1.0
bereavement from primary caregiver	NA	1.0	1.0
separation from family members	NA	1.1	1.1
mental health problems of mother	NA	1.1	1.1
mental health problems of father	NA	1.0	1.0
alcohol consumption of mother	NA	1.0	1.0
alcohol consumption of father	NA	1.0	1.0
bullied	NA	1.1	1.0
lifetime experience of suicidal thoughts at age 12	NA	1.0	1.0
Withdrawn	1.4	1.4	1.3
Somatic complaints	1.2	1.2	1.5
Anxious depressed	1.1	1.1	NA
Social problems	1.2	1.2	NA
Attention problems	1.3	1.3	NA
Aggressive behavior	1.2	1.2	NA
Persistent high Withdrawn * Increasing Somatic complaints	NA	NA	1.7

For Model 3 and Model 4, the maximum VIFs from the 100 imputed data sets were displayed for each variable.

Model 1 was a univariable logistic regression analysis examining the association between each symptom trajectory and suicidal thoughts. Model 2 was a multivariable logistic regression analysis including only symptoms that were significant in Model 1. Model 3 added confounders to Model 2. Model 4 was a multivariable logistic regression analysis including symptoms that were significant in, Model 3, their interaction term, and confounders. The 'Low' subgroup was used as a reference for all symptoms.

© 2024 Uno A et al. *JAMA Network Open.*

eTable 6. The overall effect of each symptom trajectory group

**Variable**	***p*-value**
Withdrawn	<0.0001
Somatic complaints	<0.0001
Anxious depressed	<0.0001
Social problems	0.10
Attention problems	0.0059
Delinquent behavior	0.34
Aggressive behavior	0.015

The *p*-values were obtained by a chi-square test for the likelihood of two models: one model including only the intercept and another model including a specific symptom trajectory group as an independent variable.

© 2024 Uno A et al. *JAMA Network Open.*

eAppendix 2. Guidelines for Reporting on Latent Trajectory Studies (GRoLTS) Checklist: Guidelines for Reporting on Latent Trajectory Studies

	Checklist Item	Reported?
1.	Is the metric of time used in the statistical model reported?	Yes
2.	Is information presented about the mean and variance of time within a wave?	Yes
3a.	Is the missing data mechanism reported?	Yes
3c.	Is a description provided of what variables are related to attrition/missing data?	Yes
4.	Is information about the distribution of the observed variables included?	Yes
5.	Is the software mentioned?	Yes
6a.	Are alternative specifications of within-class heterogeneity considered (e.g., LCGA vs. LGMM) and clearly documented? If not, was sufficient justification provided as to eliminate certain specifications from consideration?	Yes
6b.	Are alternative specifications of the between-class differences in variance-covariance matrix structure considered and clearly documented? If not, was sufficient justification provided as to eliminate certain specifications from consideration?	Yes
7.	Are alternative shape/functional forms of the trajectories described?	No
8.	If covariates have been used, can analyses still be replicated?	No
9.	Is information reported about the number of random start values and final iterations included?	Yes
10.	Are the model comparison (and selection) tools described from a statistical perspective?	Yes
11.	Are the total number of fitted models reported, including a one-class solution?	Yes
12.	Are the number of cases per class reported for each model (absolute sample size, or proportion)?	Yes
13.	If classification of cases in a trajectory is the goal, is entropy reported?	Yes
14a.	Is a plot included with the estimated mean trajectories of the final solution?	Yes
14b.	Are plots included with the estimated mean trajectories for each model?	Yes
14c.	Is a plot included of the combination of estimated means of the final model and the observed individual trajectories split out for each latent class?	Yes
15.	Are characteristics of the final class solution numerically described (i.e., means, SD/SE, n, CI, etc.)?	Yes
16.	Are the syntax files available (either in the appendix, supplementary materials, or from the authors)?	Yes

© 2024 Uno A et al. *JAMA Network Open.*

eAppendix 3. Sensitivity analysis of the outcome dichotomization

We used a self-report questionnaire, called 'Do you currently think that you should not be alive? to assess suicidal thoughts. The questionnaire provided the following choices: 'no', 'somewhat no', 'somewhat yes', and 'yes'. For our main analysis, we dichotomized the participants into those without suicidal thoughts (answered 'no' and 'somewhat no') and those with suicidal thoughts (answered 'yes' and 'somewhat yes'). To validate of this dichotomization, we performed a sensitivity analysis that only considered participants who answered 'yes' as having suicidal thoughts.

In this sensitivity analysis, 1.8% (35/1920) of participants had suicidal thoughts at the age of 16. The results were similar to the main analysis, with the exception of Delinquent behavior, which was found to be significant in Model 1 and included in the subsequent models.

	Model 1			Model 2			Model 3			Model 4		
	OR	95% CI	*p*-value	OR	95% CI	*p*-value	OR	95% CI	*p*-value	OR	95% CI	*p*-value
Persistent high Withdrawn	4.56	2.31-9.03	<0.0001	1.77	1.07-2.95	0.028	2.80	1.03-7.63	0.044	3.48	1.07-11.37	0.039
Increasing Somatic complaints	5.74	2.80-11.76	<0.0001	2.31	1.41-3.79	0.00093	2.62	1.02-6.72	0.046	2.19	1.14-7.80	0.026
Decreasing Anxious depressed	1.32	0.44-3.94	0.62	0.98	0.56-1.69	0.93	0.51	0.14-1.81	0.296	NA	NA	NA
Increasing Anxious depressed	5.86	2.72-12.64	<0.0001	1.98	1.15-3.43	0.015	1.24	0.41-3.76	0.706	NA	NA	NA
Persistent high Anxious depressed	2.91	0.66-12.83	0.16	0.79	0.29-2.12	0.64	0.30	0.04-2.29	0.248	NA	NA	NA
Persistent lower-middle Social problems	2.12	1.02-4.41	0.043	1.08	0.69-1.68	0.75	1.11	0.45-2.75	0.822	NA	NA	NA
Persistent upper-middle Social problems	1.80	0.52-6.23	0.35	0.68	0.31-1.52	0.35	0.49	0.09-2.62	0.405	NA	NA	NA
Persistent high Social problems	5.77	1.27-26.27	0.47	0.57	0.14-2.43	0.45	1.60	0.16-15.75	0.689	NA	NA	NA
Persistent middle Attention problems	2.29	1.10-4.74	0.027	1.07	0.67-1.72	0.78	1.52	0.59-3.92	0.390	NA	NA	NA
Persistent high Attention problems	3.30	1.20-9.10	0.021	1.42	0.63-3.21	0.40	0.85	0.16-4.42	0.850	NA	NA	NA
Persistent middle Delinquent behavior	2.62	1.17-5.88	0.019	0.68	0.37-1.25	0.21	1.16	0.39-3.48	0.79	NA	NA	NA
Persistent high Delinquent behavior	3.78	0.49-29.49	0.20	1.09	0.26-4.52	0.90	1.18	0.08-18.48	0.90	NA	NA	NA
Decreasing Aggressive behavior	2.36	1.09-5.10	0.029	1.27	0.74-2.17	0.38	1.02	0.36-2.93	0.967	NA	NA	NA
Persistent high Withdrawn * Increasing Somatic complaints	NA	NA	NA	NA	NA	NA	NA	NA	NA	0.64	0.13-3.23	0.59

OR: odds ratio, CI: confidence interval

Model 1 was a univariable logistic regression analysis examining the association between each symptom trajectory and suicidal thoughts. Model 2 was a multivariable logistic regression analysis including only symptoms that were significant in Model 1. Model 3 added confounders to Model 2. Model 4 was a multivariable logistic regression analysis including symptoms that were significant in, Model 3, their interaction term, and confounders. The 'Low' subgroup was used as a reference for all symptoms.

eAppendix 4. Additional analysis of the cross-sectional relationship between symptoms and suicidal thoughts

To examine the cross-sectional association between symptoms and suicidal thoughts, we performed logistic regression analysis using standardized CBCL subscale scores at age 16 instead of symptom trajectories in the main analysis. We fitted three models. Model 1 was a univariable logistic regression analysis for each symptom. Model 2 was a multivariable logistic regression analysis including symptoms that were significant in Model 1. Model 3 added the confounders to Model 2. Missing values were handled by multiple imputation methods. The imputation procedure included explanatory variables, outcome variables, and covariates. One hundred datasets were created and combined according to Rubin's rule.

In Model 1, all symptoms were significantly associated with suicidal thoughts. In Model 2, Withdrawn (OR 1.45, 95%CI 1.20-1.76) and Social problems (OR 0.71, 95%CI 0.53-0.95) were significantly associated with suicidal thoughts. In Model 3, Withdrawn (OR 1.57, 95%CI 1.28-1.92) and Social problems (OR 0.69, 95%CI 0.51-0.93) remained significantly associated with suicidal thoughts.

	**Model 1**			**Model 2**			**Model 3**		
	**OR**	**95% CI**	***p*-value**	**OR**	**95% CI**	***p*-value**	**OR**	**95% CI**	***p*-value**
Withdrawn	1.64	1.46-1.83	<0.0001	1.45	1.20-1.76	0.00015	1.57	1.28-1.92	<0.0001
Somatic complaints	1.37	1.25-1.50	<0.0001	1.12	0.99-1.27	0.065	1.10	0.96-1.25	0.16
Anxious depressed	1.63	1.45-1.84	<0.0001	1.23	0.99-1.53	0.060	1.09	0.86-1.37	0.48
Social problems	1.30	1.11-1.53	0.0015	0.71	0.53-0.95	0.020	0.69	0.51-0.93	0.015
Attention problems	1.46	1.27-1.69	<0.0001	1.13	0.86-1.49	0.38	1.13	0.85-1.50	0.41
Delinquent behavior	1.21	1.06-1.37	0.0038	0.92	0.75-1.12	0.40	0.90	0.72-1.13	0.37
Aggressive behavior	1.40	1.21-1.64	<0.0001	1.03	0.80-1.33	0.81	1.03	0.79-1.36	0.82

OR: odds ratio, CI: confidence interval

The VIF of the variables used in the model were all less than 3.5, suggesting that multicollinearity was minimal.

**Variable**	**Model 2**	**Model 3**
sex	NA	1.1
annual household income	NA	1.1
bereavement from primary caregiver	NA	1.0
separation from family members	NA	1.1
mental health problems of mother	NA	1.2
mental health problems of father	NA	1.1
alcohol consumption of mother	NA	1.2
alcohol consumption of father	NA	1.3
bullied	NA	1.1
lifetime experience of suicidal thoughts at age 12	NA	1.1
Withdrawn	2.7	2.7
Somatic complaints	1.7	1.7
Anxious depressed	3.0	3.1
Social problems	2.6	2.6
Attention problems	3.3	3.3
Delinquent behavior	1.9	1.9
Aggressive behavior	2.3	2.3

NA: not applicable
For Model 3, the maximum VIF in the 100 data sets of the multiple imputations for each variable was shown.

© 2024 Uno A et al. *JAMA Network Open.*

eTable 7. The raw CBCL subscale scores for each symptom trajectory

symptom	trajectory	Age 10	Age 12	Age 16
Withdrawn	Low (n=2387)	1.06 (1.17)	0.89 (1.09)	0.93 (1.17)
	Persistent high (n=393)	4.01 (2.21)	4.49 (2.28)	5.03 (2.32)
Somatic complaints	Low (n=2547)	0.42 (0.94)	0.47 (0.87)	0.77 (1.05)
	Increasing (n=233)	1.71 (1.83)	2.74 (2.15)	5.94 (2.44)
Anxious depressed	Low (n=2124)	1.59 (1.63)	1.09 (1.36)	0.99 (1.42)
	Decreasing (n=360)	7.50 (2.38)	5.17 (2.46)	2.77 (1.91)
	Increasing (n=208)	3.59 (2.03)	4.62 (2.56)	8.67 (2.89)
	Persistent high (n=88)	11.15 (3.69)	11.00 (4.02)	9.90 (4.08)
Social problems	Low (n=1869)	1.17 (1.04)	0.85 (0.94)	0.69 (0.93)
	Persistent lower-middle (n=655)	3.88 (1.35)	3.19 (1.32)	2.17 (1.46)
	Persistent upper-middle (n=213)	6.17 (1.62)	5.81 (1.63)	4.54 (1.79)
	Persistent high (n=43)	8.91 (2.20)	9.39 (1.70)	7.74 (2.23)
Thought problems	Low (n=2780)	0.52 (1.05)	0.44 (1.00)	0.50 (1.06)
Attention problems	Low (n=1912)	1.91 (1.51)	1.53 (1.36)	1.49 (1.50)
	Persistent middle (n=685)	5.86 (2.04)	5.19 (1.98)	4.65 (2.33)
	Persistent high (n=183)	9.97 (2.63)	9.53 (2.63)	8.81 (2.74)
Delinquent behavior	Low (n=2452)	0.63 (0.83)	0.44 (0.73)	0.42 (0.72)
	Persistent middle (n=301)	2.87 (1.47)	2.84 (1.50)	2.74 (1.94)
	Persistent high (n=27)	6.54 (2.52)	7.62 (2.25)	7.27 (3.30)
Aggressive behavior	Low (n=2402)	2.81 (2.67)	2.19 (2.28)	1.70 (2.18)
	Decreasing (n=378)	11.99 (4.59)	10.66 (4.89)	7.99 (4.91)

Values are means (SD).

© 2024 Uno A et al. *JAMA Network Open.*

eFigure 3. Trajectories of each somatic complaint per subgroup

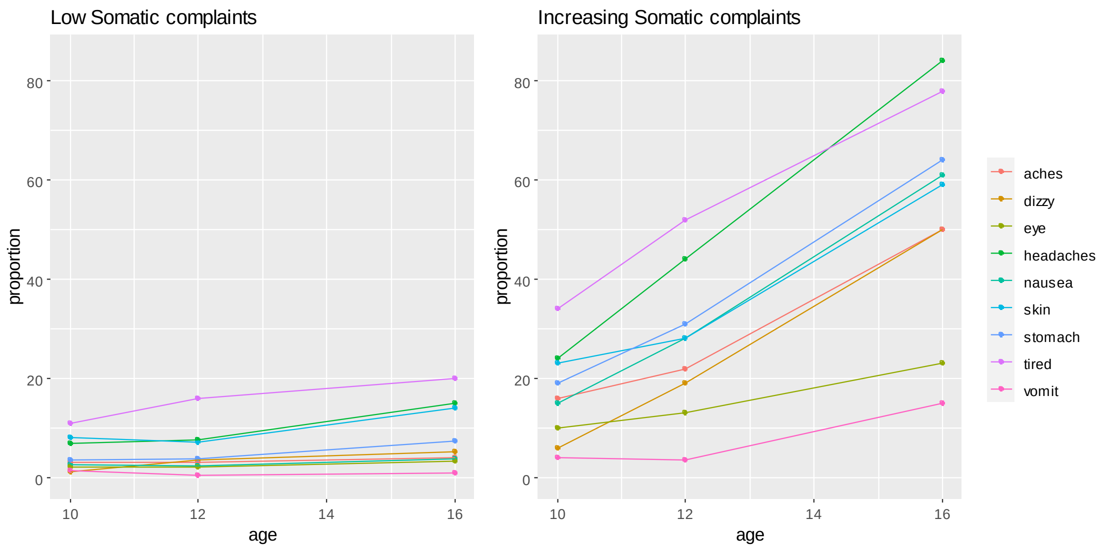

The vertical axis represents the proportion of respondents who answered 'somewhat or sometimes true' or 'very true or often true' for each symptom at each measurement point.

© 2024 Uno A et al. *JAMA Network Open.*

eReference

1. Stanyon D, Yamasaki S, Ando S, et al. The role of bullying victimization in the pathway between autistic traits and psychotic experiences in adolescence: Data from the Tokyo Teen Cohort study. *Schizophr Res*. 2022;239:111-115. doi:10.1016/j.schres.2021.11.015

2. Fujikawa S, Ando S, Nishida A, et al. Disciplinary slapping is associated with bullying involvement regardless of warm parenting in early adolescence. *J Adolesc*. 2018;68:207-216. doi:10.1016/j.adolescence.2018.07.018

© 2024 Uno A et al. *JAMA Network Open.*
