# Supplementary material for: Suicidal Thoughts and Trajectories of Psychopathological and Behavioral Symptoms in Adolescence
Source: JAMA Netw Open. 2024 Jan 25;7(1):e2353166. doi: 10.1001/jamanetworkopen.2023.53166 (PMC10811562; doi:10.1001/jamanetworkopen.2023.53166)
Supplement: Supplement 2. — Data Sharing Statement [file jamanetwopen-e2353166-s002.pdf]

Data Sharing Statement

Uno. Suicidal Thoughts and Trajectories of Psychopathological and Behavioral Symptoms in Adolescence. *JAMA Netw Open*. Published January 25, 2024.
doi:10.1001/jamanetworkopen.2023.53166

Data
Data available: Yes
Data types: Deidentified participant data
How to access data: The initial contact point for data sharing is [sandou-tky@umin.ac.jp].
When available: With publication

Supporting Documents
Document types: None

Additional Information
Who can access the data: researchers whose proposed use of the data has been approved
Types of analyses: for any purpose
Mechanisms of data availability: Data become available after the research committee approves the specific research protocol sent to them.
